# Protein disulfide isomerase-mediated apoptosis and proliferation of vascular smooth muscle cells induced by mechanical stress and advanced glycosylation end products result in diabetic mouse vein graft atherosclerosis

**DOI:** 10.1038/cddis.2017.213

**Published:** 2017-05-25

**Authors:** Suning Ping, Shuying Liu, Yuhuan Zhou, Ziqing Li, Yuhuang Li, Kefeng Liu, Adham SA Bardeesi, Linli Wang, Jingbo Chen, Lie Deng, Jingjing Wang, Hong Wang, Dadi Chen, Zhengyu Zhang, Puyi Sheng, Chaohong Li

**Affiliations:** 1 Department of Histology and Embryology, Zhongshan School of Medicine, Sun Yat-sen University, Guangzhou, Guangdong, China; 2 Department of Joint Surgery, The First Affiliated Hospital of Sun Yat-sen University, Guangzhou, Guangdong, China; 3 Experimental Center for Basic Medical Teaching, Zhongshan School of Medicine, Sun Yat-sen University, Guangzhou, Guangdong, China; 4 Department of Histology and Embryology, School of Basic Medicine, Guangzhou Medical University, Guangzhou, Guangdong, China

## Abstract

Protein disulfide isomerase (PDI) involves cell survival and death. Whether PDI mediates mechanical stretch stress (SS) and/or advanced glycosylation end products (AGEs) -triggered simultaneous increases in proliferation and apoptosis of vascular smooth muscle cells (VSMCs) is unknown. Here, we hypothesized that different expression levels of PDI trigger completely opposite cell fates among the different VSMC subtypes. Mouse veins were grafted into carotid arteries of non-diabetic and diabetic mice for 8 weeks; the grafted veins underwent simultaneous increases in proliferation and apoptosis, which triggered vein graft arterializations in non-diabetic or atherosclerosis in diabetic mice. A higher rate of proliferation and apoptosis was seen in the diabetic group. SS and/or AGEs stimulated the quiescent cultured VSMCs, resulting in simultaneous increases in proliferation and apoptosis; they could induce increased PDI activation and expression. Both *in vivo* and *in vitro*, the proliferating VSMCs indicated weak co-expression of PDI and SM-*α*-actin while apoptotic or dead cells showed strong co-expression of both. Either SS or AGEs rapidly upregulated the expression of PDI, NOX1 and ROS, and their combination had synergistic effects. Inhibiting PDI simultaneously suppressed the proliferation and apoptosis of VSMCs, while inhibition of SM-*α*-actin with cytochalasin D led to increased apoptosis and cleaved caspases-3 but had no effect on proliferation. In conclusion, different expression levels of PDI in VSMCs induced by SS and/or AGEs triggered a simultaneous increase in proliferation and apoptosis, accelerated vein graft arterializations or atherosclerosis, leading us to propose PDI as a novel target for the treatment of vascular remodeling and diseases.

Cardiovascular diseases caused by hypertension, hyperglycemia and hyperlipidemia are the leading causes of morbidity and mortality in the clinic. However, the cardiovascular diseases mainly occur in arteries rather than veins. Once veins are grafted into the arteries (e.g., vein graft bypass), their structures immediately changed and developed arterializations or atherosclerosis.^1, 2^ This might be one of the possible reasons that account for about 50% of occlusions due to increased neoimtima within the 5–10 years after coronary artery bypass surgery. Advanced glycosylation end products (AGEs) are one of the most important factors triggering vascular disease in diabetes, and the levels of AGEs significantly increase in both blood and tissues.^1^ However, the vena cava from both non-diabetic and diabetic mice in venous circulation have no pathological changes, suggesting that the increased arterial pressure-induced mechanical stretch stress (SS) has a key role in the initiation of vascular remodeling (e.g., arterializations), while diabetic-related AGEs can synergistically amplify the signals of arterializations initiated by SS, leading to the rapid formation of vein graft atherosclerosis. Thus, it is necessary to study the combination effects of SS and AGEs since the pathological processes of vascular remodeling induced by SS and AGEs separately were different from that combined. Unfortunately, until today, the reports concerning the combination of SS and AGEs were limited due to the limited published data.

Cell proliferation and apoptosis are the key players in regulating vascular homeostasis,^3^ but for a long time the majority of researchers were only focused on either proliferation or apoptosis, and few observed the proliferation and apoptosis at the same time in the same field of vision.^4, 5^ Recently, we established a new approach leading to easier observation of the proliferating, apoptotic and resting cells in the same field of view and found that simultaneous increases in proliferation and apoptosis of vascular smooth muscle cells (VSMCs) existed in the vein grafts *in vivo* and cell cultures *in vitro* in response to the same stimulation of SS and/or AGEs.^6^ But the reason why the simultaneous increases in proliferation and apoptosis of VSMCs occurred under the same stimuli remains largely unclear.

Abnormally increased SS (induced by arterial blood pressure) and AGEs (resulted from hyperglycemia) lead to the dedifferentiation of contractile VSMCs in the vessel medium into synthetic VSMCs;^7^ similarly the adventitial fibroblasts or undifferentiated mesenchymal stem cells can also be differentiated into synthetic VSMCs.^8, 9, 10, 11^ Although the cultured VSMCs are derived from the same artery and belong to the synthetic VSMCs, further cell phenotypic differences can be obtained after cell passages, leading to the heterogeneity of the structures in the synthetic cells.^12^ The contractile VSMCs de-differentiate into synthetic VSMCs accompanying decreased contractile organelles, for example, myofilament, dense body and patch, and increased synthetic organelles, for example, rough endoplasmic reticulum and Golgi complex. SM-*α*-actin is one of the most important components of myofilaments. Recently, we found that different levels of SM-*α*-actin expression in VSMCs are closely associated with the selective activation of three MAPK members, implying the simultaneous increase in the proliferation and apoptosis in response to the same extracellular stimuli, such as SS and AGEs,^6^ but which molecules mediate SS- and/or AGEs-induced simultaneous increase in the proliferation and apoptosis needs to be determined.

The redox system is an important intracellular signal system that is capable of affecting cell structure and function. PDI is one of the members of the thioredoxin superfamily oxidoreductases, which includes about 20 subtypes,^13^ in this system.^13, 14, 15^ PDI gene knockout is lethal to cells and embryos.^16^ PDI has three main functions: molecular chaperones, oxidoreductase and isomerase^13, 17, 18^ and can promote the disulfide formation (oxidase), breakage (reductase) or rearrangement (isomerase) of oxidized proteins, which are directly involved in the regulation of endoplasmic reticulum stress,^15, 19^ oxidative stress,^20^ resulting in the occurrence and development of many human major diseases, such as cancer, neurological degenerative changes, immune and viral infections and infertility.^14, 19, 21, 22, 23^ PDI inhibition prevents an increase in the reduced form of PDI in human neuroblastoma SH-SY5Y cells treated by 1-methyl-4-phenylpyridinium (MPP^+^), suppresses excessive protein folding, endoplasmic reticulum stress and induces clearance of aggregated *α*-synuclein in Parkinson’s disease by increased autophagy.^24^ It has been reported that the expression levels and activity of PDI in the heart tissue of patients with hyperglycemia has been changed.^25^ However, no data directly address whether the expression and activation of PDI in VSMCs are closely associated with the simultaneous increases in proliferation and apoptosis and whether VSMC phenotypes or subtypes in response to the same extracellular stimuli can influence it.

In our preliminary experiments we found that SS and AGEs can induce increased oxidized PDI (ox-PDI) with different expression levels, which modulates NOX1 expression. Here, we hypothesized that different expression levels of PDI mediate completely opposite cell fates among the different VSMC subtypes in response to SS and/or AGEs partially via NOX/ROS signaling. This study provides new insight into understanding the molecular mechanisms of formation of vein graft atherosclerosis and a novel strategy for the prevention and treatment of vascular remodeling diseases.

## Results

### PDI upregulation promotes simultaneous increases in proliferation and apoptosis leading to diabetic vein graft atherosclerosis

**Figure 1:**
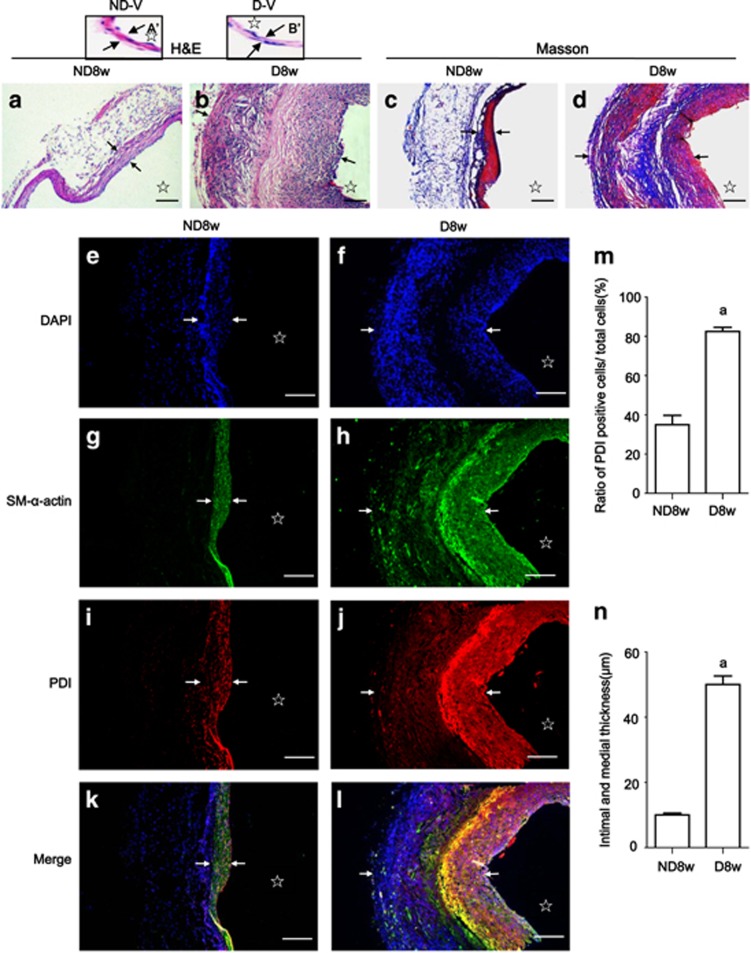
PDI upregulation in smooth muscle cells leads to accelerated diabetic vein graft arteriosclerosis. Paraffin-embedded sections of the vein grafts of non-diabetic and diabetic mice killed 8 weeks after surgery were used for: (**a**, **b**) H&E staining shows the wall structures in vein grafts of non-diabetic and diabetic mice and the respective vena cava (A’, B’) were used for the control of the vein grafts; (**c, d**) Masson trichromatic staining shows the wall structures and deposited collagenous fibers (blue color) in the vein grafts of non-diabetic and diabetic mice; (**e–l**) triple-labeling immunofluorescence with primary PDI, SM-alpha-actin antibodies and FITC, or Cy3-conjugated secondary antibody was performed and counterstained with DAPI; (**e, f**) DAPI staining shows cell nuclei (blue) in the walls of vein grafts from non-diabetic and diabetic mice; (**g, h**) SM-*α*-actin positive cells (green) show VSMCs in the vein grafts from non-diabetic and diabetic mice; (**i, j**) the cells with PDI expression (red) in the vein grafts from non-diabetic and diabetic mice; (**k, l**) merged images show co-expression (yellow) of PDI (red) and SM-alpha-actin (green) in VSMCs in the walls of vein grafts from (**e** to **j**). (**m**) Statistical results show ratios of PDI-positive cell numbers/ total cell numbers from (**e, f and i, j**) from three independent experiments; (**n**) statistical results show wall thickness of vein grafts from (**a, b**) from third independent experiments. **a**, *P*<0.05 *versus* non-diabetic mice as indicated by analysis of variance (ANOVA) with LSD test. Data are shown as means±SEM. Scale bar, 100 *μ*m. Arrowheads and stars indicate the VSMCs thickness and lumens of the vein grafts, respectively

Histopathological analysis with H&E or Masson staining was performed to determine the effects of increased blood pressure and high glucose on vascular remodeling. The walls of the vein grafts in both groups appeared to be thicker than the respective vena cava (Figures 1A’ and B’),^1^ but the vein grafts of diabetic mice showed more profound abnormalities in the vessel layers than those from the non-diabetic mice (Figures 1a and b). Deposited collagen fibers were mainly found in the medium of the vein grafts of diabetic mice (Figures 1c and d).

Immunofluorescent analysis was performed to explore the relationship among PDI, SM-*α*-actin expression and vein graft remodeling. Either 1 row of cells (DAPI stained) in the vein grafts of the non-diabetic mice or two rows of cells in the diabetic mice were found. Between the two rows of cells in the diabetic vein grafts there was an akaryotic area (Figures 1e–l) in which cells with strong co-expression of SM-*α*-actin (Figures 1g, h, k and l) and PDI (Figures 1i–l) could be found, indicating that akaryotic cells with a strong expression of SM-*α*-actin in the medium were dead VSMCs, which were closely associated with PDI overexpression while the cells in the medium of the non-diabetic vein grafts (Figure 1e) and the neointima and adventitia of the diabetic vein grafts (Figure 1f) represented weakly co-expressed SM-*α*-actin and PDI (Figures 1g–l), implying that strong co-expression of SM-*α*-actin and PDI closely associated with cell death and weak co-expression of both relates to cell proliferation.

**Figure 2:**
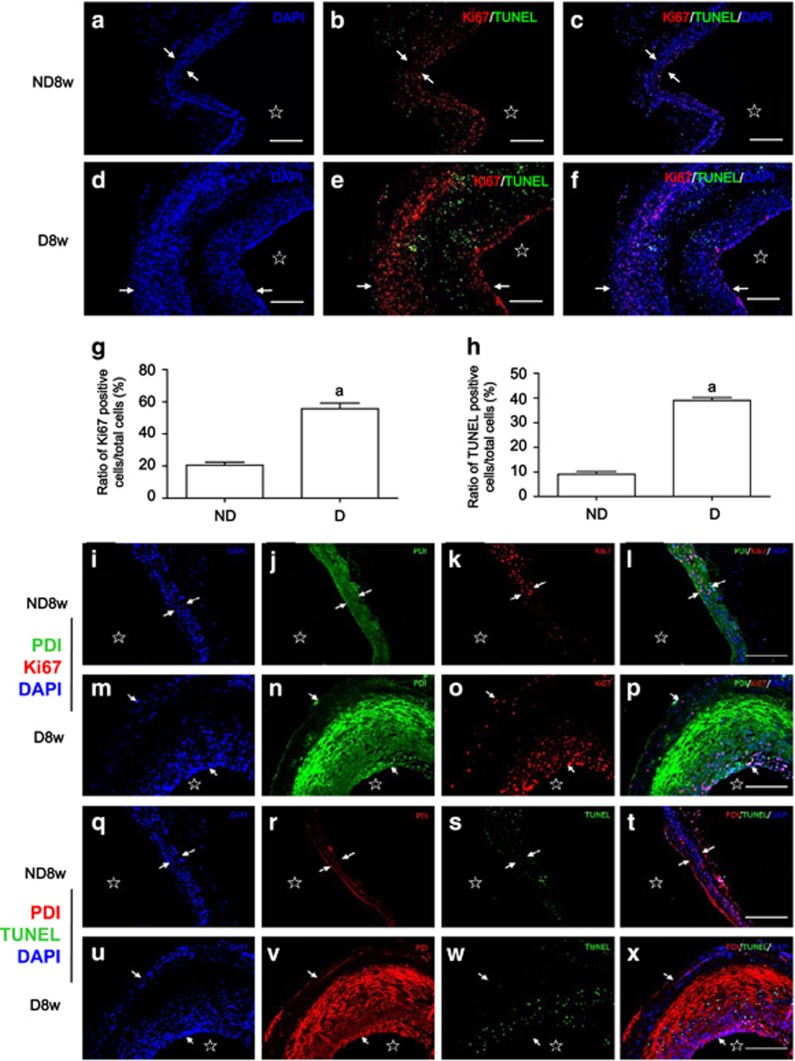
PDI upregulation promotes simultaneous increases in proliferation and apoptosis in the diabetic vein graft atherosclerosis. Paraffin-embedded sections of the vein grafts of non-diabetic and diabetic mice killed 8 weeks after surgery were used for: (**a–f**) triple-labeling immunofluorescence with primary Ki67 antibody and TUNEL kit and Cy3-conjugated secondary antibody was performed and counterstained with DAPI; (**a, d**) DAPI staining shows cell nuclei (blue) in the walls of vein grafts from non-diabetic and diabetic mice. An akaryotic area can be seen through the medium of the diabetic vein grafts; (**b, e**) merged images from Ki67-positive (red) and TUNEL-labeling (green) cells in the walls of vein grafts from non-diabetic and diabetic mice; (**c, f**) Merged images show co-distribution of proliferating cells (red, Ki67-positive) and apoptotic cells (green, TUNEL-labeling) in the walls of vein grafts from (**a, b, d** and **e**); (**g, h**) statistical results show ratios of Ki67- and TUNEL-positive cell numbers/ total cell numbers from (**a** to **f**) and from three independent experiments. a, *P*<0.05 *versus* non-diabetic mice as indicated by analysis of variance (ANOVA) with LSD test. Data are shown as means±SEM. (**i–x**) Triple-labeling immunofluorescence with primary PDI and Ki67 antibodies or TUNEL kit and Cy3-conjugated secondary antibody was performed and counterstained with DAPI. (**i, m, q and u**) DAPI staining shows cell nuclei (blue) in the walls of vein grafts from non-diabetic and diabetic mice. (**j, n, and r, v**) PDI staining (**j, n,** green; and **r, v,** red**)** in the cells of vein grafts from non-diabetic and diabetic mice; (**k, o**) Ki67-positive cells (red, proliferating cells) and (**s**, **w**) TUNEL-positive cells (green, apoptotic cells) in the vein grafts from non-diabetic and diabetic mice; (**l**, **p**) Merged images show co-distribution of PDI-labeling cells (green) and proliferating cells (red, Ki67-positive) and (**t, x**) PDI-labeling cells (red) and apoptotic cells (green, TUNEL-labeling) in the walls of vein grafts from non-diabetic and diabetic mice. Scale bar, 100 *μ*m. Arrowheads and stars indicate the VSMCs thickness and lumens of the vein grafts, respectively

Ki67 has been widely used as a biomarker of cell proliferation. Triple fluorescent staining with Ki67 antibody and TUNEL kit in the presence of DAPI was used to simultaneously observe the distribution of proliferating and apoptotic cells in the vein grafts. Ki67-positive cells (proliferating cells; Figures 2a–h) were mainly found in both neointima and adventitia in the walls of the diabetic vein grafts and in the medium of the vein grafts of the non-diabetic mice, while many TUNEL-positive cells (apoptotic cells; Figures 2a–h) near the akaryotic VSMCs were visible in the medium of the diabetic vein grafts. Ki67-positive cells were mainly located in the neointima and adventitia of the diabetic vein grafts and close to the TUNEL-positive cells, forming a cycle, in which the dead cell induces apoptosis, and then apoptosis induces proliferation. Also, mixed distribution of Ki67- and TUNEL-positive cells could be seen in the neointima and adventitia of the diabetic vein grafts. Some resting cells were visible in the proliferating areas in both groups (Figures 2a–h). The results suggest that the simultaneous increases in proliferation and apoptosis of VSMCs are common events during both arterializations of the non-diabetic vein grafts and arteriosclerosis of the diabetic vein grafts.

The relationship between the expression of PDI and proliferation or apoptosis in vein grafts was explored (Figures 2i–x). Results demonstrated that very strong PDI expression was observed in the akaryotic cells and apoptosis-positive cells in the diabetic vein grafts (Figures 2u–x), while a weak expression of PDI was observed in the proliferating areas in both groups (Figures 2i–p). These findings further confirmed that strong co-expression of PDI and SM-*α*-actin in cells may contribute to the death of VSMCs, and weaker co-expression of PDI and SM-*α*-actin leads to VSMC proliferation (Figures 1e–l and Figures 2a–x). Arterial blood pressure alone can induce weak PDI expression in the VSMCs of the vein grafts. This can be synergistically enhanced by diabetes-related AGEs, leading to an increased PDI expression in VSMCs accelerating vein graft arteriosclerosis.

### SS and/or AGEs activate PDI in VSMCs *in vitro*

**Figure 3:**
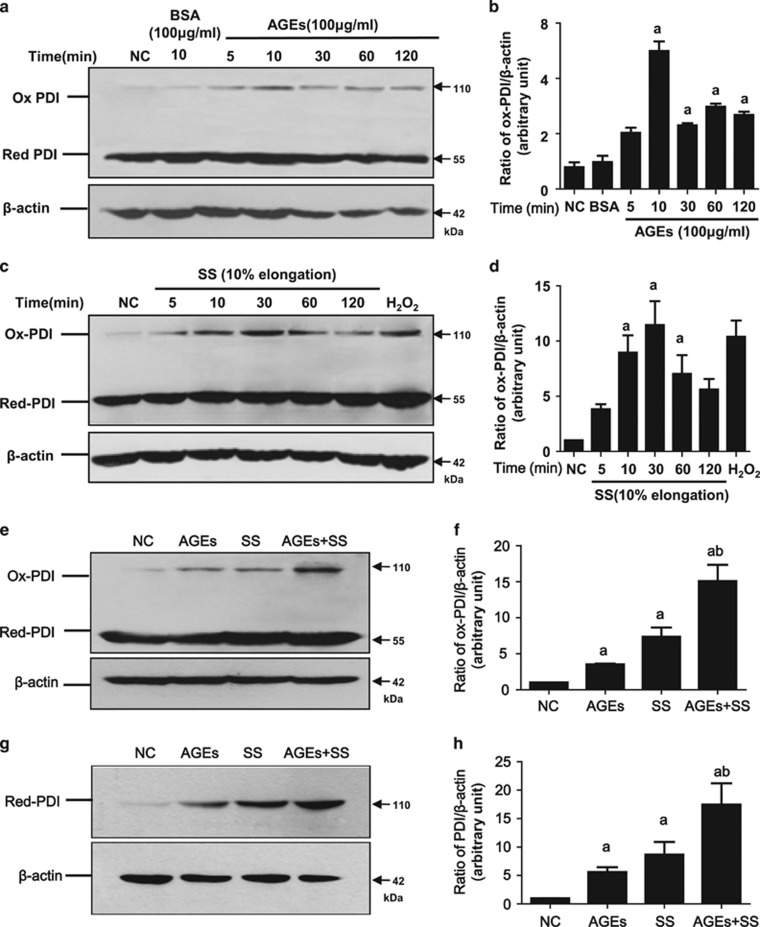
SS and AGEs activate PDI in VSMCs *in vitro*. 70% confluent VSMCs were serum-starved for 24 h and treated with SS, AGEs or both for 10 min (SS, 10% elongation and indicated time) and (AGEs, 100 *μ*g/ml)or for 1 h, and cultured for additional 23 h, and harvested for detection of ox-PDI or total PDI by western blot, respectively. (**a, c and e**) SS and/or AGEs-induced increases of ox-PDI in VSMCs; (**g**) SS and/or AGEs-induced increases of total PDI in VSMCs. Beta-actin was set as internal loading control; (**b, d, f** and **h**) statistical results of ratios of ox-PDI/ or total PDI/Beta-actin from (**a**, **c**, **e** and **g**) from three independent experiments. a, *P*<0.05 *versus* negative control (NC); b, P<0.05 *versus* AGEs or stretch stress (SS) by analysis of variance (ANOVA) with LSD test. Data are shown as the means±SEM

**Figure 4:**
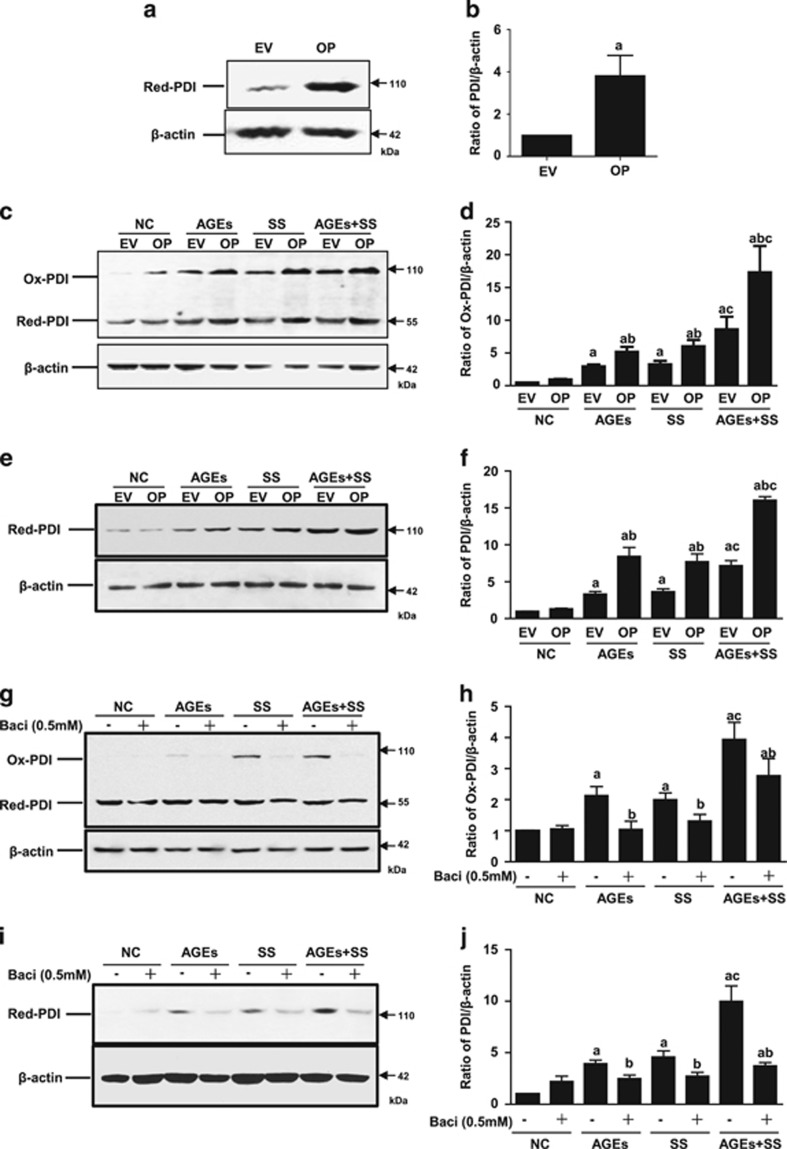
The effects of PDI overexpression or PDI inhibitor on changed ox-PDI and total PDI in VSMCs induced by SS and/or AGEs. (**a**–**f**) VSMCs lines with stable PDI overexpression were identified by western blot and then used for experiments of SS and/or AGEs-induced ox-PDI or total PDI analysis; (**g**–**j**) quiescent cultured VSMCs pretreated with PDI inhibitor (bacitracin, 0.5 mM) for 1 h were treated with SS, AGEs or both for 10 min, and harvested for ox-PDI analysis or for 1 h, cultured additional 24 h, and then harvested for total PDI analysis by western blot. Beta-actin was set as an internal control; (**b, d, f, h** and **j**) statistical results of ratios of ox-PDI/ or total PDI/beta-actin from (**a, c, e, g** and **i**) from three independent experiments, respectively. a, *P*<0.05 *versus* negative control (NC); b, *P*<0.05 *versus* AGEs or SS of the same group; c, *P*<0.05 *versus* AGEs or SS of the different group by ANOVA with LSD test. Data are shown as means±SEM

An increase in PDI oxidation (ox-PDI) is thought to be PDI activation, which causes intracellular signal transduction. To investigate the effects of increased arterial blood pressure-induced SS and hyperglycemia-induced AGEs on PDI activation (the increase of oxidized PDI, ox-PDI) and expression, quiescent VSMCs were treated with SS and/or AGEs. Both AGEs and SS alone could induce the rapid activation of PDI (increased ox-PDI) in a time-dependent manner (Figures 3a–d). SS and AGEs induced an increase of ox-PDI as early as 5 min, peaked at 30 min, and then declined. Combined treatment with both induced more ox-PDI than the sum of the single treatments (Figures 3e and f). Prolonged treatment with SS, AGEs, or both triggered a greater increase of total amounts of PDI (Figures 3g and h). Stable overexpression of PDI in VSMCs also resulted in more ox-PDI induced by SS, AGEs or both when compared to PDI-empty vector VSMCs (Figures 4a–f). Prolonged treatment with SS, AGEs or both led to increased total PDI expression (Figures 4e and f). In contrast, inhibition of PDI using an inhibitor (bacitracin) led to less production of ox-PDI and decreased total PDI expression (Figures 4g–j). These results suggest that the PDI participates in the intracellular signal transduction of VSMCs induced by SS and AGEs via oxidized/reduced forms, and combined stimulation by both SS and AGEs may further promote PDI activation synergistically.

### PDI modulates NOX1 expression in VSMCs

**Figure 5:**
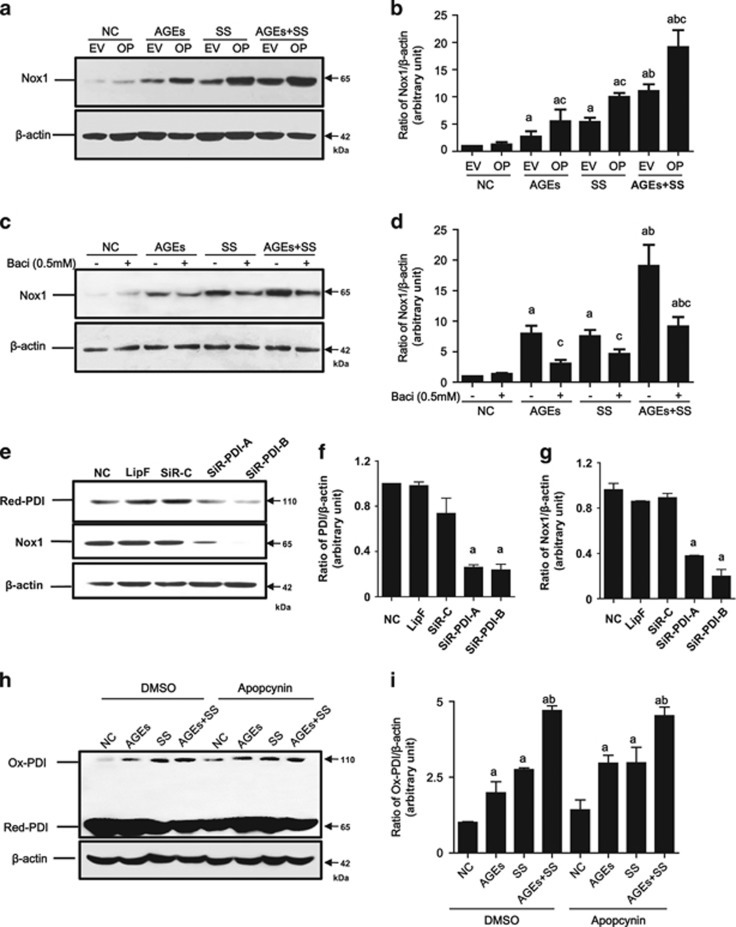
The effects of overexpression, inhibitor and SiRNA of PDI and NADPH oxidases inhibitor on changed total Nox1 in VSMCs induced by SS and/or AGEs. (**a**, **b**) The quiescent cultured VSMCs with stable PDI overexpression were treated with SS, AGEs or both for 1 h and continually cultured for additional 24 h; (**c**, **d**) The quiescent cultured VSMCs pretreated with DMSO (NC) or PDI inhibitor bacitracin (PDI-I) for 1 h were treated by SS and/or AGEs for 1 h and continually cultured for additional 24 h; (**e–g**) the cultured VSMCs transfected with siRNA-PDI-A, and -B (SiR-PDI-A and -B), siRNA-control (SiR-C) and lipofectamine (Lip); (**h**, **i**) the quiescent cultured VSMCs pretreated with DMSO (NC) or NADPH oxidase inhibitor (apopcynin, 0.1 mM) for 1 h, and then treated by SS and/or AGEs for 10 min. All treated cells above were then harvested to detect total PDI and/or Nox1or ox-PDI by Western blot. Beta-actin was set as an internal control. (**b**, **d**, **f**, **g** and **i**) Statistical results of ratios of total NOX1, PDI or ox-PDI/beta-actin from (**a, c, e** and **h**) from three independent experiments, respectively. **a**, *P*<0.05 *versus* negative control (NC); b, *P*<0.05 *versus* AGEs or SS of the same group; **c**, *P*<0.05 *versus* cells without PDI overexpression or without inhibitors of each group by ANOVA as indicated by LSD test. Data are shown as means±SEM

To evaluate the relationship between PDI and NOX1 in VSMCs, PDI overexpression cell lines and the PDI inhibitor (bacitracin) were used. Compared to VSMCs with PDI-empty vector, PDI overexpression induced significant increases in NOX1, which was found to be further enhanced by treatment with SS, AGEs or both (Figures 5a and b). In contrast, treatment of VSMCs with the PDI inhibitor and siRNA-PDI suppressed the expression of the total PDI, which also triggered decreases in the NOX1 expression (Figures 5c–g). However, VSMCs pretreated with an inhibitor (apocynin) of nicotinamide-adenine dinucleotide phosphate oxidases (NADPH oxidases) demonstrated no effect on either the total PDI expression or ox-PDI (Figures 5h and i), but did decrease NOX1 expression. These results indicate that NOX1 may have a role as a downstream molecule of PDI induced by SS and/or AGEs.

### PDI mediates SS and/or AGE-induced production of ROS via NOX1 signaling

**Figure 6:**
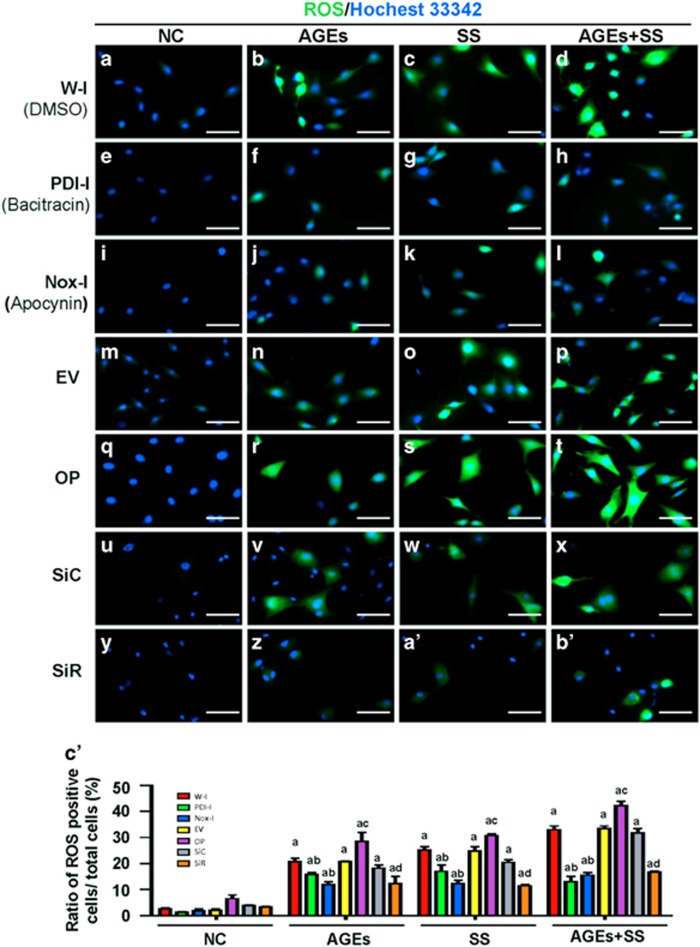
The effects of inhibitor, overexpression, SiRNA of PDI and NOX inhibitor on ROS production in VSMCs induced by SS and/or AGEs. The quiescent cultured VSMCs were treated with SS and/or AGEs for 10 min after pretreatment with DMSO (NC) (**a**–**d**), PDI inhibitor (bacitracin, PDI-I) (**e–h**), and NADPH oxidase inhibitor (apocynin, NOX-I) (**i**–**l**) for 1 h. The quiescent VSMCs with stable PDI overexpression or VSMCs transfected with siRNA-control (SiR-C) (**u–x**) or siRNA-PDI (SiR-PDI) (**y**, **z**, **a’**, **b’**) were treated by SS and/or AGEs for 10 min. All treated cells above were stained with the reactive oxygen species (ROS) probe (green) and then counterstained with Hochest 33342 (blue). ROS-positive cells are shown in green and the nuclei of VSMCs in blue. (**c’**) Statistical results of ratios of ROS-positive cells were obtained from (**a**–**z**, **a’**, **b’**) of three independent experiments. **a**, *P*<0.05 versus negative control (NC); **b**, *P*<0.05 versus without inhibitors of the same group; **c**, *P*<0.05 versus cells without PDI overexpression (EV); **d**, *P*<0.05 versus SiC by ANOVA with LSD test. Data are shown as the means±SEM

NADPH oxidases in vascular cells are one of the main sources of ROS, which have an important role in cell proliferation and apoptosis. The effects of PDI and NADPH oxidases on the production of ROS in response to SS, AGEs and both were evaluated. SS and AGEs alone induced increases in ROS in VSMCs, and the combination treatment with both triggered a synergistic increase in ROS levels (Figures 6a–d). However, PDI stable overexpression in VSMC lines could further amplify the effects mentioned above on ROS production when compared to VSMCs with empty vectors (6M-P). In contrast, separate treatment of VSMCs with the PDI inhibitor (bacitracin) (Figures 6e–h), NADPH oxidase inhibitor (apocynin; Figures 6i–l), or siRNA-PDI (Figures 6y, z, a’ and b’) was found to inhibit SS and/or AGE-induced ROS production. These findings suggest that PDI and NOX1 mediate ROS production in VSMCs in response to SS, AGEs or both.

### PDI mediates simultaneous increases in proliferation and apoptosis of VSMCs induced by SS, AGEs or both

**Figure 7:**
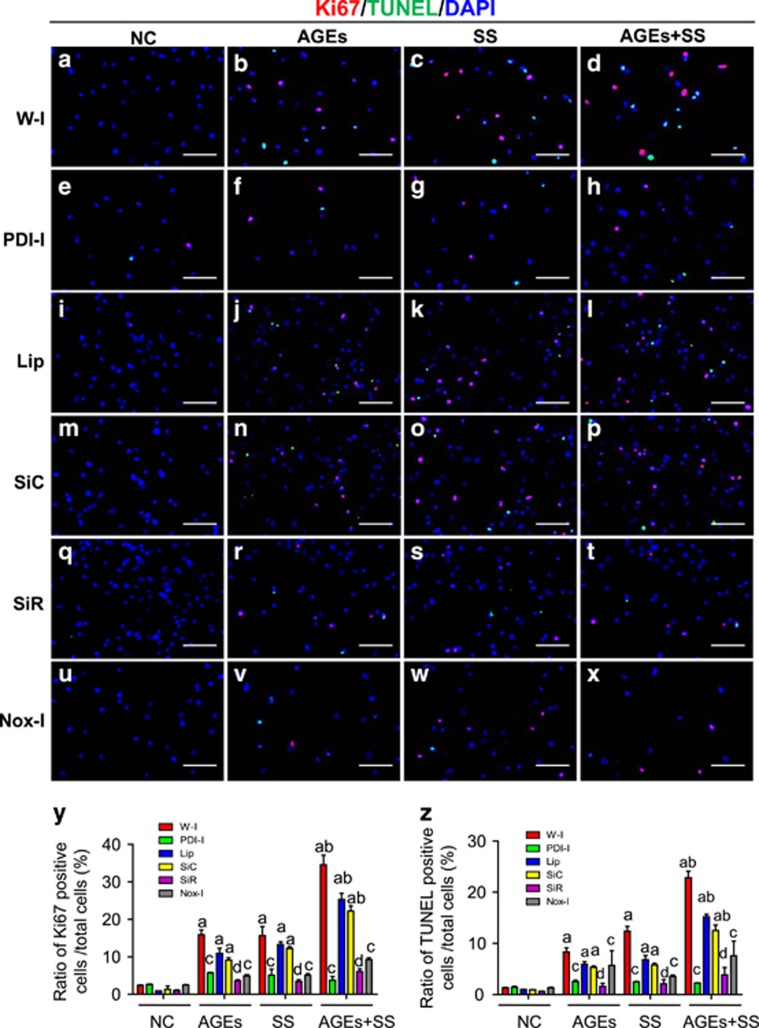
The effects of PDI inhibitor, PDI-SiRNA and NADPH oxidase inhibitor on simultaneous increases in proliferation and apoptosis of VSMCs induced by SS and/or AGEs. The quiescent cultured VSMCs were pretreated with DMSO (**w–i**) (**a**–**d**) or PDI inhibitor bacitracin (**e**–**h**), or NADPH oxidase inhibitor (apocynin) (**u**–**x**) for 1 h, or transfected with SiRNA-PDI (SiR) (**m**–**p**), siRNA-control (SiC) (**q**–**t**) and Lipofectamine (Lip) (**i**–**l**). All cells above pretreated or not were subjected to SS and/or AGEs for 1 h and continually cultured for additional 24 h. These cells were stained with the primary Ki-67 antibody, Cy3-conjugated secondary antibody, and a TUNEL kit and then counterstained with DAPI. Ki-67-positive cells are shown in red, TUNEL-positive cells in green, and the nuclei of VSMCs in blue. (**y**, **z**) Statistical results of the ratio of Ki-67- or TUNEL-positive cells from **a** to **x** were obtained from three independent experiments. **a**, *P*<0.05 versus negative control (NC); **b**, *P*<0.05 versus SS or AGEs; **c**, *P*<0.05 versus without inhibitors of the same group; **d**, *P*<0.05 versus SiC of the same group by ANOVA with LSD test. Scale bar, 100 *μ*m. Data are shown as the means±SEM

The effects of PDI on the simultaneous increases in the proliferation and apoptosis induced by SS, AGEs or both have not been reported. Here, quiescent VSMCs were pretreated with inhibitors of PDI or NADPH oxidases, and cell proliferation and apoptosis at the same view were observed. Compared to the negative control (Figure 7a), both SS and AGEs alone could induce simultaneous increases in the proliferation and apoptosis of VSMCs (Figures 7b and c), and the combination stimulation with both led to greater increases in proliferation and apoptosis in VSMCs (Figures 7d). In contrast, the PDI inhibitor (bacitracin) (Figures 7e–h) significantly suppressed the simultaneous increases in the proliferation and apoptosis of VSMCs induced by SS, AGEs and both. Analogously, siRNA-PDI treatment also led to significant decreases in the proliferation and apoptosis (Figures 7q–t) relative to the siRNA-control (Figures 7m–p). Apocynin (NADPH oxidase inhibitor) (Figures 7u–x) also demonstrated partial inhibition of simultaneous increases in the proliferation and apoptosis of VSMCs induced by SS, AGEs and both. These results suggest that PDI may mediate simultaneous increases in proliferation and apoptosis of VSMCs induced by SS, AGEs, or both via the downstream molecules, for example, NADPH oxidases-ROS signaling.

### Subtypes of synthetic VSMCs determine expression levels of PDI and proliferation or apoptosis of VSMCs induced by SS, AGEs or both

**Figure 8:**
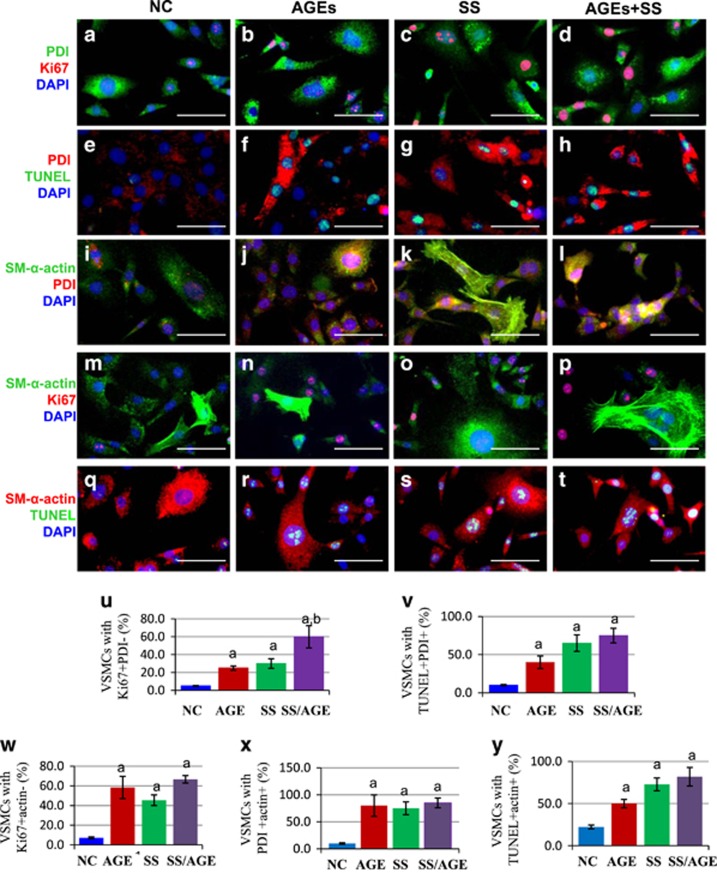
Subtypes of VSMCs determine expression levels of PDI and proliferation or apoptosis of VSMCs induced by SS, AGEs or both. The quiescent cultured VSMCs were subjected to SS and/or AGEs for 1 h, and continually cultured for additional 24 h. These cells were then stained with primary PDI and Ki-67 antibodies, FITC or Cy3-conjugated secondary antibodies, and a TUNEL kit, and then counterstained with DAPI. (**a–d**) The triple-labeling immunofluorescent shows Ki-67-positive (red), and PDI-labeling cells (green). (**e–h**) TUNEL-positive (green) and PDI-labeling cells (red). (**i–l**) SM-*α*-actin positive (green) and PDI-positive cells (red). (**m–p**) SM-*α*-actin positive (green) and Ki67-positive cells (red); (**q–t**) SM-*α*-actin positive (red) and TUNEL-positive cells (green). Using ImageJ program, the cells were sorted into two groups, cells with low and negative PDI or SM-*α*-actin expression and high and strong PDI or SM-*α*-actin expression. Then the Ki67-positive or TUNEL-positive cells were counted, respectively. DAPI stained cells were used for total cell numbers. (**u** and **v**) Statistical results of the ratio of Ki-67- or TUNEL-positive cells and PDI from (**a** to **d**) and (**e** to **h,**) respectively; (**w** and **y**) show statistical results of the ratio of Ki-67- or TUNEL-positive cells and SM-*α*-actin from (**m** to **p**) and (**q** to **t**), respectively; (**x**) shows statistical results of the ratio of PDI and SM-*α*-actin from (**i** to **l**). These statistical results were obtained from three independent experiments. a, *P*<0.05 *versus* negative control (NC); and b, *P*<0.05 *versus* SS or AGEs. Scale bar, 100 *μ*m. Data are shown as the means±SEM

The cultured and passaged VSMCs belong to synthetic VSMCs, but their expression levels of SM-*α*-actin are different, which are termed as subtypes of synthetic VSMCs. To investigate the molecular mechanisms by which simultaneous increases in the proliferation and apoptosis were induced by SS and/or AGEs, we performed *in situ* simultaneous observations of PDI, SM-*α*-actin, proliferation and apoptosis. The cells with weak PDI or SM-*α*-actin expression represented more as being Ki67-positive, and with less apoptotic labeling, and strong PDI or SM-*α*-actin expression indicated more apoptotic positive and less Ki67 labeling (Figure 8). In addition, co-expression of PDI and SM-*α*-actin expression could be confirmed as shown in Figures 8i–l.

To uncover the molecular mechanisms by how SM-*α*-actin determines the proliferation or apoptosis of VSMCs induced by SS, AGEs or both, an inhibitor of actin (cytochalasin D) was used to pretreat the quiescent VSMCs, and then SS, AGEs or both stimulated the cells. Analysis via western blot indicated that compared to the negative control, SS, AGEs or both slightly induced the decrease of SM-*α*-actin expression, while cytochalasin D led to remarkable inhibition of SM-*α*-actin expression (Supplementary Figures 1A and B). SS, AGEs or both significantly induced the increase of cleaved caspase-3. Surprisingly, cytochalasin D further promoted the activation of caspase-3, not inhibited it (Supplementary Figures 1C and D). Activation of caspase-3 enhanced the induction of cell apoptosis, which was further confirmed by triple-stained immunofluorescence (Supplementary Figure 2). Compared to the negative control, single cytochalasin D induced cell apoptosis. SS, AGEs, or both all triggered simultaneous increases in proliferation and apoptosis, while apoptosis was further enhanced by the treatment of cytochalasin D, but there was no effect on proliferation. These results suggest that VSMC apoptosis seems to be SM-*α*-actin-dependent in response to SS, AGEs or both.

## Discussion

Based on a new approach recently established and data observed by us,^6^ in this study, we further proposed the novel idea that different expression levels of PDI in VSMCs mediate the simultaneous increases in proliferating and apoptotic cells *in vivo* and *in vitro* induced by SS and/or AGEs. Our data indicate the following: (1) the simultaneous increases in proliferating, apoptotic cells can be seen in the same view of vein grafts and cultures induced by SS and AGEs and are required for vein graft arterializations in non-diabetic mice and atherosclerosis in diabetic mice; (2) the activation and different expression of PDI across the individual VSMCs induced by SS and/or AGEs leads to the simultaneous increases in VSMC proliferation and apoptosis; (3) VSMC subtypes characterized by various SM-*α*-actin expression respond differently to the same extracellular stimuli triggering the simultaneous increases in proliferation and apoptosis via PDI/NOX/ROS signaling; (4) veins from the mice themselves (both the non-diabetic and diabetic mice) have no changes in structure, but the veins grafted into arteries change their structures soon after operation; and (5) VSMC apoptosis is SM-*α*-actin-dependent via the caspase-3 signal pathway. Together, these data significantly extended our current knowledge concerning vascular remodeling and diseases, and provided an important experimental platform for studying the underlying mechanisms of tissue and organ remodeling involved in cell proliferation and apoptosis, such as organ transplantation, cancer therapy, embryo and fetal development, and so on. Importantly, they may make PDI a new potential target for the prevention and treatment of vascular remodeling and diseases.^26^

Cell proliferation and apoptosis have key roles in vascular remodeling, including atherosclerosis and vein graft arteriosclerosis. Most of the research data have demonstrated that vascular remodeling is mainly caused by increased VSMC proliferation and decreased VSMC apoptosis after vessel injury. However, these data primarily resulted from single observations on proliferation or apoptosis. In the present study, we found that the collagen fiber-like structures denoted by HE-staining located in the medium of the diabetic vein grafts are the dead VSMCs, not collagen fibers (Figure 1). These dead VSMCs strongly co-express SM-*α*-actin and PDI, suggesting a close relationship between the dead VSMCs and PDI. The grafted veins were getting thicker in both groups accompanying simultaneous increases in proliferation and apoptosis compared with the mouse veins themselves. Proliferating VSMCs in non-diabetic graft veins mainly located in media and only a small part of apoptotic cells were found in the adventitia. However, the wall thickness in the diabetic vein grafts was more obvious than that in the non-diabetic vein grafts. Of the proliferating VSMCs primarily located in the neointima and adventitia, among them were some apoptotic cells distributed diffusely. Interestingly, many akaryotic dead VSMCs could be seen in the media. In the periphery of this akaryotic zone, there was a large number of dying apoptotic cells, which were surrounded by the proliferating cells, forming a ‘dead cell-inducing apoptosis, apoptotic cell-inducing proliferation’ vicious circle, leading to accelerated vascular remodeling and eventually diseases. Apoptotic cells can release mitogenic signals to stimulate neighboring cell proliferation for increasing tissue repair and regenerative growth;^27, 28^ however, apoptotic VSMCs in the vessel walls are highly differentiated cells and hard to remove due to long myofilaments in the cytoplasm. These results indicate that the excessive inducing apoptosis of VSMCs seems to be deleterious for vascular tissue repair or remodeling. Several model organisms (e.g., hydra, planarians, newts, and mice) concerning apoptosis-inducing proliferation have been established.^27, 28^ Here, we first reported the observation results in vascular remodeling and diseases. Although either SS or AGEs could induce simultaneous increases in VSMC proliferation and apoptosis *in vitro*, no structural changes in the vena cava of both the normal and diabetic mice *in vivo* could be found (Figures 1A’ and B’), further addressing the significance of increased SS induced by arterial pressure during vein graft remodeling. SS is very important, but single mechanical stress induced by increased arterial blood pressure is insufficient to cause apoptosis-inducing proliferation in the non-diabetic vein grafts, which can be synergistically induced by AGEs in the diabetic vein grafts, suggesting the pathological processes of vascular remodeling induced by single or a combination of SS and AGEs are different. Therefore, studying the role of the combination of SS and AGEs is necessary. Salzberge *et al.* first established diabetic vein graft mouse models, and found that the neointima of the grafted veins were much thicker in diabetic than non-diabetic mice due to the increase in the extracellular matrix. However, they have not observed the obvious proliferation of vascular cells, and also did not detect apoptosis.^29^ Kalra *et al.* found the increase of proliferation and apoptosis in veins grafted into the carotid arteries of dogs, but their data only resulted from separate slices. So neither the relationship between proliferation and apoptosis nor the pathogenic vascular changes in diabetics was clear.^30^

In our research, we took the advantage of analyzing the influence and distribution of proliferation and apoptosis both *in vivo* and *in vitro* under the same conditions. Our results suggest that increased SS-induced by arterial pressure plays key roles in the initiation of vascular remodeling, and AGEs induced by high blood glucose can synergistically amplify the SS-initiated signals to accelerate the formation of diabetic vein graft causing atherosclerosis through a mechanism referred to as dead cell-inducing apoptosis and apoptosis-inducing proliferation.

Why do these cells have completely opposite fates under the same stimuli? What are the key players in regulating the process? We recently reported that three MAPK members (e.g., ERKs, JNKs, and p38MAPK) are selectively activated, and closely associated with SM-*α*-actin expression in VSMCs. However, regardless of short- or long-term stimulation of SS, AGEs or both, makes the intracellular phosphorylation of MAPKs change, and the total amount of MAPK protein does not change, implying that MAPKs are not the key player in regulating the process. However, data indicate that PDI is directly involved in the regulation of endoplasmic reticulum stress^15, 19^ and oxidative stress,^20^ resulting in the development of cancer, neurological degenerative changes, immune and viral infections, and infertility.^14, 19, 21, 22, 23^ In this study, we for the first time connected PDI with the simultaneous increases in VSMC proliferation and apoptosis *in vitro* and *in vivo* and confirmed different PDI expression across individual VSMCs (Figure 8). The VSMCs with strong PDI expression were mainly akaryotic dead and dying apoptotic cells, while the cells with weak PDI expression preferentially were proliferating cells (Figures 1, 2 and 8). Either SS or AGEs could activate PDI (increased ox-PDI), and upregulate PDI expression to further induce increases of NOX1 expression and ROS production. The combined SS and AGEs had synergistic effects. Studies from other groups have shown that intracellular PDI regulates the expression and activity of the NADPH oxidase family of proteins (Nox), which are enzymes dedicated to ROS generation.^31^ PDI can closely interact with Noxes, and support growth factor-dependent Nox1 activation and mRNA expression, as well as migration in smooth muscle cells. PDI overexpression induces acute spontaneous Nox activation.^20^ ROS can have important roles as signaling molecules to serve useful physiological functions and to induce very harmful effects by causing oxidative damage.^32^ In the present study, after PDI inhibition or silencing, the increases of NOX1 expression, ROS production, proliferation, and apoptosis of VSMCs were simultaneously inhibited, which means that PDI can mediate the simultaneous increases in proliferation and apoptosis induced by SS and/or AGEs via partially regulating the oxidative stress pathway.

PDI could be immediately activated and upregulated to different expression levels in single cells exposed to SS and AGEs. SM-*α*-actin expression in VSMCs was slightly influenced by SS and AGEs via western blot analysis (Supplementary Figure 1), but the co-expression profiles of SM-*α*-actin and PDI were similar (Figure 8). These results suggest that PDI is a key mediator in regulating cell proliferation and apoptosis, while the subtypes of VSMCs have a key role in determining different PDI expressions to induce various NOX1 expression and ROS production, which lead to completely opposite cell fates (Figure 8 and Supplementary Figure 1). Cells with strong co-expression of SM-*α*-actin and PDI were more prone to apoptosis and the cells with weak co-expression of SM-*α*-actin and PDI were easier to proliferate in response to same stimuli. Our *in vivo* results (Figures 1 and 2) are fully consistent with the *in vitro* results (Figures 6, 7, 8). The convincing evidence is that different cell types and subtypes have been found in grafted veins such as endothelium in the intima, VSMCs in media, fibroblasts or undifferentiated mesenchymal stem cells in the adventitia and migrated inflammation cells.^33, 34, 35, 36^ VSMCs have important roles in the vascular remodeling and include contractile, synthetic VSMCs and other unidentified subtypes. These different phenotypes or subtypes of VSMCs can be related to the different reactions in response to the same stimuli.^37^ Our recent data showed that different subtypes of VSMCs lead to selective activation of three MAPK members, accelerating vein graft atherosclerosis in response to SS and/or AGEs. VSMCs with strong SM-*α*-actin expression demonstrate activation of P38MAPK and JNKs leading to apoptosis, while the weak expression of SM-*α*-actin triggers ERKs activation resulting in proliferation.^6^ In addition, the primary cultured cells can also be divided into different phenotypes or subtypes after several passages.^38^ The expression levels of SM-*α*-actin across individual cells are quite different.^6^ These results suggest that there are close relationships between SM-*α*-actin and the PDI/NOX/ROS pathway and proliferation and apoptosis.

Muscle cells have three types of actins, including *α*-, *β*- and *γ*-actins. They constitute the main composition of the cytoskeleton in the cell, which handles the functions of cell contraction, motility, vesicle trafficking, intracellular organization, cytokinesis and endocytosis.^39^ Some researchers found actin to be a sensor and inducer of apoptosis.^40^ Caspase-3-induced gelsolin fragmentation contributes to actin cytoskeleton collapse, nucleolysis and apoptosis of VSMCs exposed to proinflammatory cytokines.^41^ Actin can have roles as a substrate of caspases and be cleaved into two small fragments of 31 kDa (F-actin) and 14 kDa (t-actin).^3^ T-actin was found to induce apoptotic cell characteristics; cell transfection with T-actin rather than F-actin can also demonstrate apoptosis. Deletion of protein kinase C*δ* in VSMCs of mice triggers actin disorganization leading to the retardation of cell migration^42^ and the increase of cell proliferation.^43^ VSMCs with strong co-expression of SM-*α*-actin and PDI are more prone to apoptosis, while VSMCs with weak co-expression of SM-*α*-actin and PDI are more prone to proliferation (Figures 2 and 8). Treatment of cells with cytochalasin D leads to actin cytoskeleton collapse or disorganization, which increases more substrates of caspases-3, leading to promoted apoptosis (Supplementary Figures 1 and 2). SS and/or AGEs slightly decrease actin via actin disorganization, triggering morphological change, which then activates caspase-3 promoting apoptosis.^1^ It is also interesting that ROS products across VSMCs represented three levels: strong, weak and negative in response to SS and/or AGEs (Figure 6), which will be worthy of further studies in the future.

In conclusion, we for the first time provided the evidence that simultaneous proliferation and apoptosis are closely associated with VSMC subtypes via PDI/NOX/ROS signaling, a common pathway in the process of vein graft arterializations and atherosclerosis. SS induced by increased blood pressure non-specially activates the transmembrane proteins including RAGE, which can be specifically activated by AGEs induced by diabetes.^1, 6, 44, 45^ Either SS or AGEs promotes simultaneous proliferation and apoptosis, and both combinations have a synergistic effect (Supplementary Figure 3).^1, 6^ The VSMCs with strong co-expression of SM-*α*-actin and PDI are more prone to apoptosis, while VSMCs with a weak co-expression of SM-*α*-actin and PDI are more prone to proliferation. These results may make PDI a novel target for the prevention and treatment of vascular remodeling and diseases^26^ and widen our present knowledge for understanding molecular mechanisms of vein graft atherosclerosis, especially in the diabetic setting.

## Materials and methods

### Mouse models of vein grafting

All experimental procedures were similar to the reported paper^46^ with slight modifications.^1^ Three-month-old male C57BL/6 J mice were purchased from the Laboratory Animal Center of Sun Yat-sen University (Guangzhou, China), maintained on a light/dark (12/12 h) cycle at 25 °C, and received food and water ad libitum before the experiments. The mice were divided into a non-diabetic group (ND) and a diabetic group (D) (*N*=50, respectively). In the diabetic group, each mouse received seven consecutive daily injections of STZ (Sigma, St. Louis, MO, USA) (i.p. 50 mg/kg), while mice in the non-diabetic group were injected with citrate buffer as a control. Blood glucose levels of the two groups were measured 1 week later. Levels above 288 mg/dl were considered to be indicative of diabetes. Then, the mice were subjected to vein graft surgery as reported previously.^1^ In brief, the vena cava of the isogenic donor mouse was grafted into the dissected right common carotid artery of the recipient mouse. Vigorous pulsations confirmed successful engraftment. The mouse was anesthetized with sodium pentobarbital (i.p. 50 mg/kg). At the same time, atropine sulfate was also administrated at a dose of 1.7 mg/kg to keep the respiratory tract clear by reducing salivary secretions. A 10-min interval was allowed between the injection and operation to ensure that the intensity and frequency of the muscle tension was reduced. The surgery was completed within 40 min to relieve the pain. After the surgery, the recovery time was variable, ranging from 30 min to 2 h. Warm blankets and oxygen inhalation were applied to the mice. Sufficient food and water were supplied to the mice. All animal procedures were consistent with the National Institutes of Health’s Guide for the Care and Use of Laboratory Animals and approved by the Animal Care and Use Committee of Sun Yat-sen University. The vein grafts were harvested 8 weeks after the operations and were fixed with 4% paraformaldehyde. Paraffin-embedded samples were made into 4-*μ*m-thick sections for further analysis.

### *In situ* immunofluorescent staining of vein grafts

Paraffin-embedded sections were subjected to immunofluorescent staining based on the protocols provided by Abcam (www.abcam.com/technical) with slight modifications. Briefly, the sections were deparaffinized with xylene and ethanol. Antigen retrieval was then performed using Tris/EDTA buffer for 3 min in a pressure cooker. The samples were washed with TBS, permeabilized, and blocked. The sections were incubated with primary antibodies, Ki67, and PDI antibodies (1:200, Santa Cruz, CA, USA), SMC-*α*-actin antibody (1:600, Sigma) mixed in 0.3% Triton X-100 overnight at 4 °C, followed by incubation with CY3- and FITC-conjugated secondary antibodies (1:200, Jackson Immuno Research Laboratories Inc, West Grove, PA, USA) for 2 h at 37 °C. The nuclei were counterstained with 4, 6-diamidino-2-phenylindole (DAPI, 10 mg/ml in PBS) and the sections were stained using a TUNEL kit (Roche, Basel, Switzerland). Then, the samples were inspected and photographed using fluorescence microscopy(Leica DMI4000B, Leica Microsystems, Wetzlar, Germany).

### Cell culture and treatment

VSMCs were isolated by enzymatic digestion of the aortas of C57BL/6 J mice using a modified version of a previously described procedure.^47^ The isolated cells grown in gelatin coated six-well culture plates with a silicone elastomer-bottom were maintained in a humidified atmosphere of 5% CO_2_ with growth medium (DMEM+10% fetal calf serum+100 *μ*m streptomycin +100 U/ml penicillin). Cells achieving 80% confluence were serum-starved for 24 h (quiescent cultured VSMCs) and subjected to SS with Cyclic Stress Unit (Flexcell International Corp., Burlington, NC, USA) in the absence or presence of AGEs according to the procedures previously described.^1, 48^ Cyclic Stress Unit, a modification of the unit initially described by Banes *et al.*^49^ consisted of a controlled vacuum unit and a base plate to hold the culture plates (FX3000 AFC-CTL, Flexcell International Corp., Burlington, NC, USA). A vacuum (15–20 kPa) was repeatedly applied to the elastomer-bottomed plates via the base plate, which was placed in a humidified incubator with 5% CO_2_ at 37 °C. Cyclic deformation (60 cycles per min) and 10% elongation of the elastomer-bottomed plates were used. This model of the apparatus generated a homogeneous SS on the membrane. Preparation and identification of AGEs were similar to our previous report.^50^ Pretreatment of the cells with inhibitors (bacitracin for PDI, apopcynin for NADPH oxidase, and cytochalasin D for actin) (Sigma) were respectively utilized to determine the effects of these intracellular molecules on signal pathways and cell functions.

### VSMC transfection

For cDNA transfection, 0.5–2 × 10^5^ cells were plated in 500-*μ*l-growth medium without antibiotics. Cells were transfected after they reached 90–95% confluence. For each transfection sample, 0.8 *μ*g of PDI cDNA and 1 *μ*l of Lipofectamine 2000 (Life Technologies, Carlsbad, CA, USA) in Opti-MEM were incubated with cells for 24 h at 37 °C in a CO_2_ incubator. To construct stable cell lines, cells were passaged at 1:10 dilution into fresh growth medium 24 h after transfection. Selective medium containing 40 mM of G418 was added to indicate resistant monoclonal cells suitable for the corresponding experiments.

### RNA interference

VSMCs were plated in 500 *μ*l growth medium without antibiotics. Cells were transfected after they reached 30~50% confluence. The procedures used for this experiment were similar to those previously described.^51^ The PDI small interfering RNA (siRNA-PDI) targeted duplex sequences (Sense: 5′-GCCUGAGAUUCGCUAGCAAGG-3′ Antisense: 5′-CCUUUGCUAGCGAAUCUCAGAGCC-3′) were synthesized by OriGene Technologies, Inc. (Rockville, MD, USA). A non-targeting siRNA duplex sequence (universally scrambled) served as a negative control. Transfection was performed according to the manufacturer’s instructions. Serum-starved quiescent VSMCs were subjected to cyclic stress in the presence and absence of AGEs. After transfection, the samples were collected for western blot, ROS detection and immunofluorescent staining.

### Western blot analysis

PDI is sensitive to the reduction-oxidation (redox) state *in vitro*. To determine the exact redox state of the PDI, care was taken to prevent thiol-disulfide exchange reactions from taking place during and after cell lysis. In the experiment, procedures were similar to those described previously, with slight modifications.^52^ The cultured VSMCs pretreated with or without inhibitors were subjected to SS (10% elongation) and/or AGEs (100 *μ*g/ml) and then incubated with 0.2 M iodoacetamide buffer on ice. After incubation, cells were collected in lysis buffer containing protease inhibitors. The lysis suspension was centrifuged, and protein concentration was assessed using a Bio-Rad protein assay. Heat-denatured proteins were resolved using SDS-PAGE and electrophoretically transferred to nitrocellulose membranes. To maintain the redox state of the PDI, all buffers were free of dithiothreitol (DTT). Then, the membranes were probed with antibodies against PDI, NOX1 and *β*-actin antibodies. The bands were visualized using an enhanced chime-luminescence (ECL) detection system. The intensity was quantitated using Image J program (NIH Image, Bethesda, MD, USA) for densitometry.

### Reactive oxygen species detection

70–80% confluent VSMCs were serum-starved for 24 h and subjected to cyclic SS (10% elongation for 10 min) in the presence and absence of AGE. After washing with the medium, the cells were stained using H2DCFDA probe in the medium at 37 °C in a humidified atmosphere of 5% CO_2_ for 30 min. The nuclei were counterstained with Hoechst 33342 (16 *μ*mol/l in medium) after being washed with medium.^53^ Then, the samples were inspected and photographed using fluorescence microscopy (Leica DMI4000B, Leica Microsystems).

### *In situ* detection of proliferating, apoptotic and resting VSMCs in cultures

*In situ* proliferation and apoptosis of cultured VSMCs were examined by immunofluorescent staining with Ki67 antibody (Santa Cruz Biotechnology, Inc.) and a TUNEL kit (Roche Diagnostics Ltd.) in the presence of DAPI. Briefly, the treated cells (SS, 10% elongation and/or AGEs, 100 *μ*g/ml, for 1 h, and continuously cultured for an additional 24 h) were stained with the Ki67 antibody and corresponding CY3-conjugated secondary antibody (Jackson Immune Research), then with the TUNEL kit according to the kit’s instructions. The nuclei were counterstained with DAPI. The proliferating and apoptotic cells were identified by Ki67- and TUNEL-positive staining. Total nuclei (DAPI staining) and Ki67- or TUNEL-positive cells were counted and analyzed by two independent researchers blinded to the specimen groups. The proliferating and apoptotic index was calculated as the percentage of active proliferating or apoptotic cells *versus* the total cell count. Except for proliferating and apoptotic cells, all remaining cells (DAPI staining only) were considered to be resting cells. For the detection of SM-*α*-actin and PDI expression, the treated cells above were simultaneously incubated with primary PDI and Ki-67 antibodies, FITC or Cy3-conjugated secondary antibodies and then counterstained with DAPI. For the detection of SM-*α*-actin or PDI expression and proliferation, the treated cells above were simultaneously incubated with SM-*α*-actin or ki67 antibodies, and then corresponding secondary antibodies. For the detection of SM-*α*-actin or PDI expression and apoptosis, the treated cells above were simultaneously incubated with SM-*α*-actin, or ki67 antibody, and then corresponding secondary antibody and TUNEL kit according to the kit’s instructions. Nuclei were also counterstained with DAPI. The samples were inspected and photographed using fluorescence microscopy (Leica DMI4000B, Leica Microsystems). Using the ImageJ program, the cells were sorted into 2 groups: cells with low and negative PDI or SM-*α*-actin expression and high and strong PDI or SM-*α*-actin expression. Then the Ki67- or TUNEL-positive cells were counted, respectively. DAPI stained cells were used for total cell numbers.

### Statistical analysis

Statistical analysis was performed using the SPSS 13.0 package (IBM Corporation, NY, USA) for Microsoft Windows (Microsoft Corporation, WA, USA). Continuous variables are expressed as mean and SEM. One-way ANOVA was used for continuous variables and *χ*^2^ and Fisher exact tests were used for categorical variables. *P*-value<0.05 was considered statistically significant.
