# Supplementary figures and images for: Protein disulfide isomerase-mediated apoptosis and proliferation of vascular smooth muscle cells induced by mechanical stress and advanced glycosylation end products result in diabetic mouse vein graft atherosclerosis
Source: Cell Death Dis. 2017 May 25;8(5):e2818–. doi: 10.1038/cddis.2017.213 (PMC5520728; doi:10.1038/cddis.2017.213)

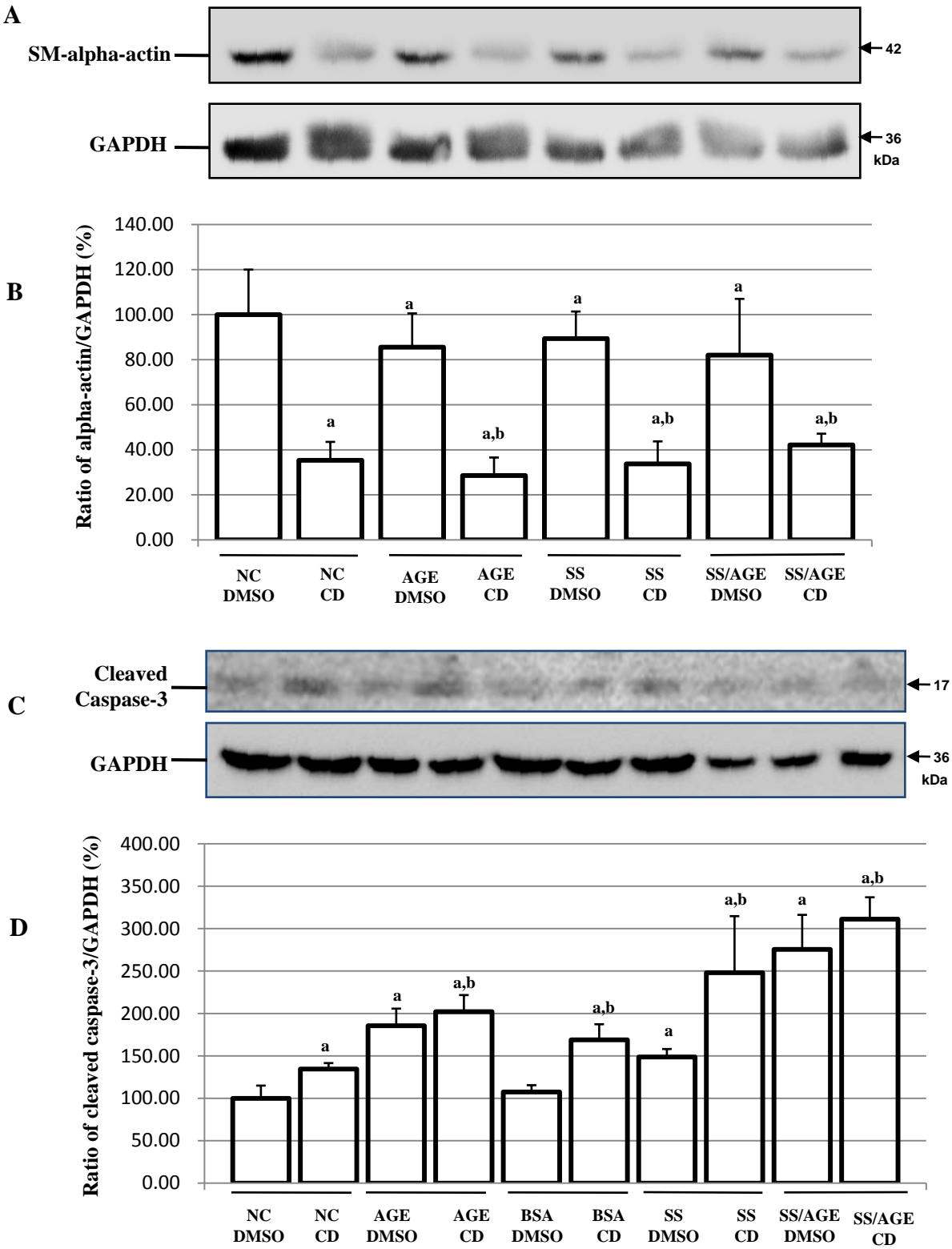

Supplement: Supplementary Figure 1 [file cddis2017213x1.pdf]

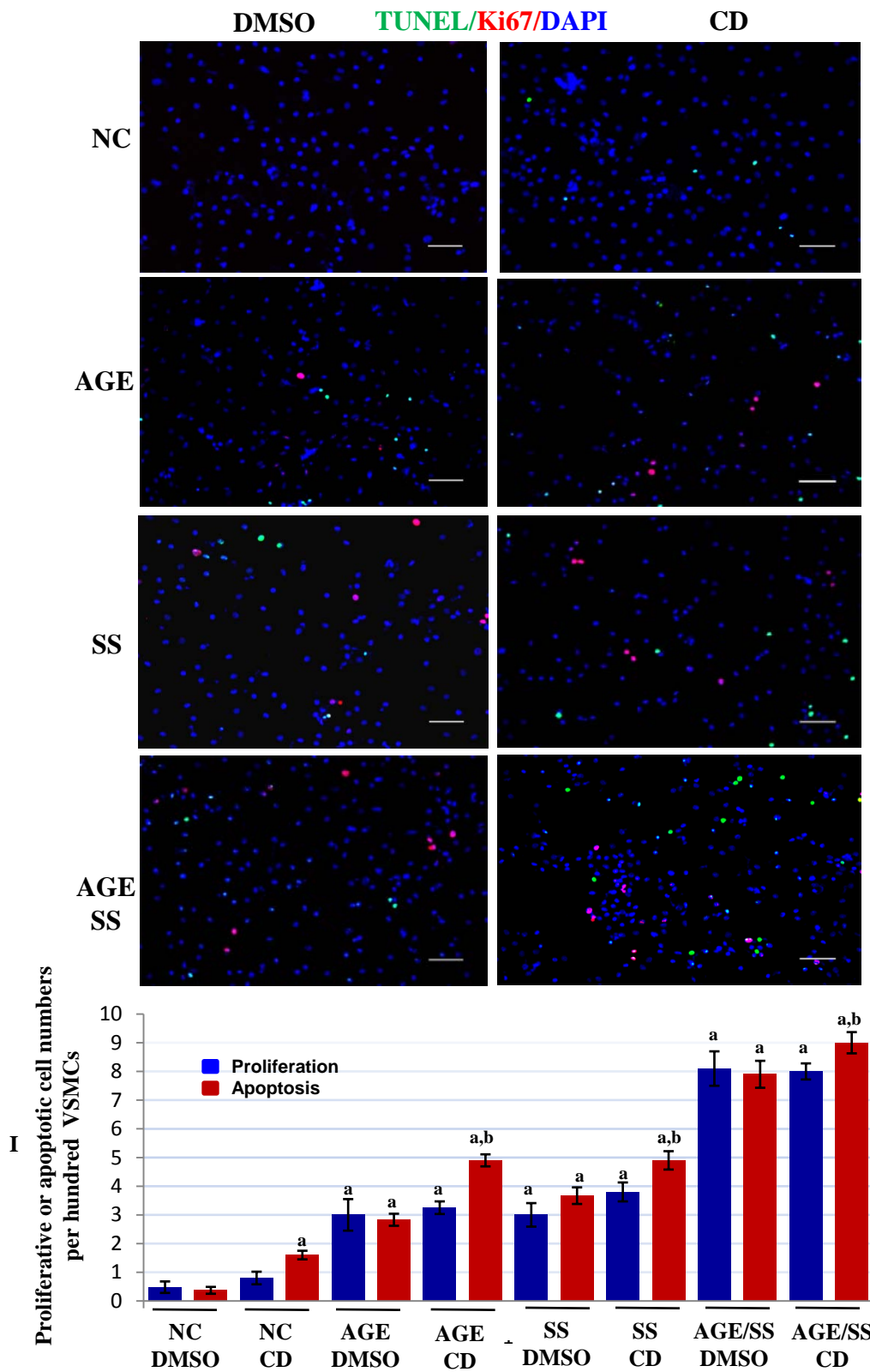

Supplement: Supplementary Figure 2 [file cddis2017213x2.pdf]

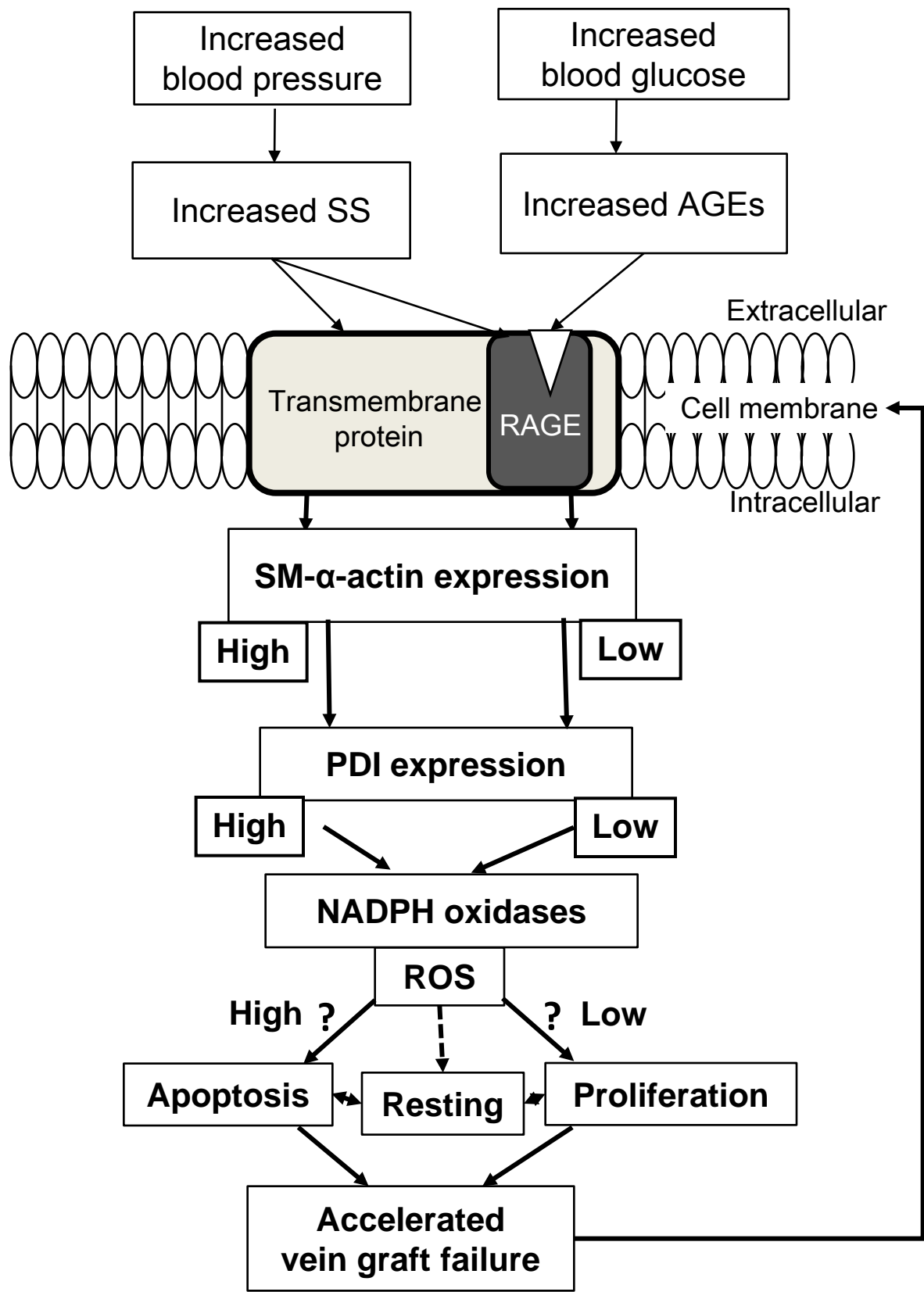

→ Single signaling  
→ Combined signaling

Supplement: Supplementary Figure 3 [file cddis2017213x3.pdf]
