# Supplementary material for: Protein disulfide isomerase-mediated apoptosis and proliferation of vascular smooth muscle cells induced by mechanical stress and advanced glycosylation end products result in diabetic mouse vein graft atherosclerosis
Source: Cell Death Dis. 2017 May 25;8(5):e2818–. doi: 10.1038/cddis.2017.213 (PMC5520728; doi:10.1038/cddis.2017.213)
Supplement: Supplementary Figure Legends [file cddis2017213x4.docx]

**Supplementary Figure 1. Cytochalasin D suppresses actin expression and activates caspase-3 in VSMCs in response to SS and/or AGEs.** The quiescent cultured VSMCs were pretreated with cytochalasin D (CD) or DMSO for 1 h, and then treated by SS and/or AGEs for 1h and cultured for additional 24h. All treated cells above were then harvested to detect SM-α-actin (**A and B**) and cleaved caspase-3 (**C and D**) by Western blot. GAPDH was set as an internal control. **B and D:** statistical results of ratios of SM-α-actin/ or cleaved caspase-3/GAPDH from **A, and C** from 3 independent experiments, respectively. a, *P*<0.05 *vs.* negative control (NC); b, *P*<0.05 *vs.* NC or AGEs or stretch stress (SS) of the same group. Data are shown as means±s.e.m.

**Supplementary Figure 2. Cytochalasin D induces VSMC apoptosis in response to SS and/or AGEs.** The quiescent cultured VSMCs were pretreated with DMSO or cytochalasin D (CD) for 1 h, and then treated by SS and/or AGEs for 1h and cultured for additional 24h. The cells were stained with the primary Ki-67 antibody, Cy3-conjugated secondary antibody, and a TUNEL kit and then counterstained with DAPI. Ki-67-positive cells are shown in **red,** TUNEL-positive cells in **green**, and the nuclei of VSMCs in **blue**. **I**, statistical results of the ratio of Ki-67- or TUNEL-positive cells from **a–h** were obtained from 3 independent experiments. a, *P*<0.05 *vs.* negative control (NC); b, *P*<0.05 *vs.* stretch stress (SS) or AGEs. Bar=100μm. Data are shown as the means±s.e.m.

**Supplementary Figure 3.** **Potential signal pathway of PDI-NOX-ROS in VSMCs induced by SS and AGEs leading to simultaneous increases in proliferation and apoptosis.** The increase in blood pressure triggers a rapid increase in mechanical stretch stress (SS) on the walls of vein grafts. SS causes deformation of the vascular cells (VSMCs) and non-specifically activates all receptors in the membrane and its downstream signal molecules, including PDI and NADPH oxidases, thus produces reactive oxygen species (ROS), which causes increased proliferation (Ki-67 positive) of the vascular cells and increased apoptosis (TUNEL positive). Hyperglycemia can produce numerous AGEs deposited on the vascular wall, where they directly and specifically interact with receptor of AGEs (RAGE) to activate intracellular signaling molecules, altering vascular structure and function. However, different levels of PDI up-regulation closely depend on levels of SM-α-actin expression in VSMCs. Further investigation may identify how the different levels of SM-α-actin expression in VSMCs determine PDI up-regulation and simultaneous increases in proliferation and apoptosis in response to SS and/or AGEs.
